# PD03 - Household smoking and markers of atopic sensitisation in children: a systematic review and meta-analysis

**DOI:** 10.1186/2045-7022-4-S1-P3

**Published:** 2014-02-28

**Authors:** Wojciech Feleszko, Marek Ruszczyński, Joanna Jaworska, Agnieszka Strzelak, Bartłomiej M Zalewski, Marek Kulus

**Affiliations:** 1 The Medical University of Warsaw, Warsaw, Poland

## Background

Household smoking (HS) is linked in children with development and aggravation of allergic asthma. Its influence on children’s immune responses and development of allergy has not been conclusively elucidated.

## Objective

To perform a systematic review evidence of HS on markers of allergic sensitization in children and adolescents.

## Methods

CENTRAL, MEDLINE, and EMBASE databases were searched in November 2012. Included studies compared environmental tobacco smoke-exposed and non-exposed children and fulfilled criteria to define objective markers of atopic sensitization defined as total immunoglobulin E concentrations (tIgE), presence of specific IgE (sIgE+), and positive skin-prick tests (SPT+). A standardized protocol was used for data extraction. Resulting data were analyzed by methods of fixed or random-effect model, and generic inverse variance analysis (RefMan software).

## Results

8 studies of HS influence on tIgE concentration (2,603 children), 6 studies on HS and sIgE+ (9,844 children) and 14 studies of ETS and SPT (14,176 children) met the preset criteria of inclusion. Parental tobacco smoking was shown to raise tIgE concentrations by average of 27.7 IU/ml (95%CI: 7.8-47.7) and to boost the risk of atopic sensitization, as assessed by sIgE+ (OR=1.12, 95%CI: 1.00-1.25) and SPT+ (OR=1.13; 95%CI: 1.02-1.26). According to a subgroup analysis, this effect became evident in the preschool children (<7 y) (OR=1.20; and OR=1.33 for sIgE+ and SPT+, respectively).

## Conclusions

This analysis underscores the association between HS in early childhood and augmented risk of allergic sensitization, and that early age of children is related to its harmful immune-modulating effects of parental tobacco smoking. Furthermore this study highlight again the role of environmental tobacco smoke exposure as the most important, avoidable risk factors for development of allergy in children.

